# The Efficacy of Psychological Interventions for Managing Fatigue in People With Multiple Sclerosis: A Systematic Review and Meta-Analysis

**DOI:** 10.3389/fneur.2018.00149

**Published:** 2018-04-04

**Authors:** Aung Zaw Zaw Phyo, Thibaut Demaneuf, Alysha M. De Livera, George A. Jelinek, Chelsea R. Brown, Claudia H. Marck, Sandra L. Neate, Keryn L. Taylor, Taylor Mills, Emily O’Kearney, Amalia Karahalios, Tracey J. Weiland

**Affiliations:** ^1^Neuroepidemiology Unit, Centre for Epidemiology and Biostatistics, Melbourne School of Population and Global Health, The University of Melbourne, Melbourne, VIC, Australia; ^2^Biostatistics Unit, Centre for Epidemiology and Biostatistics, Melbourne School of Population and Global Health, The University of Melbourne, Melbourne, VIC, Australia

**Keywords:** fatigue, multiple sclerosis, CBT, review, meta analysis

## Abstract

**Background:**

Multiple sclerosis (MS) is a complex, demyelinating disease of the central nervous system. Fatigue is commonly reported by people with MS (PwMS). MS-related fatigue severely affects daily activities, employment, socioeconomic status, and quality of life.

**Objective:**

We conducted this systematic review and meta-analysis to determine whether psychological interventions are effective in managing fatigue in PwMS.

**Data sources:**

We performed systematic searches of Medline, EMBASE, PsycINFO, and CINAHL to identify relevant articles published from database inception to April 5, 2017. Reference lists from relevant reviews were also searched.

**Study selection and design:**

Two independent reviewers screened the papers, extracted data, and appraised the included studies. A clinical psychologist verified whether interventions were psychological approaches. A narrative synthesis was conducted for all included studies. For relevant randomized controlled trials that reported sufficient information to determine standardized mean differences (SMDs) and 95% confidence intervals (CIs), meta-analyses were conducted using a random-effects model.

**Results:**

Of the 353 identified articles, 20 studies with 1,249 PwMS were included in this systematic review. Narrative synthesis revealed that psychological interventions reduced fatigue in PwMS. Meta-analyses revealed that cognitive behavioral therapy decreased levels of fatigue compared with non-active controls (SMD = −0.32; 95% CI: −0.63 to −0.01) and compared with active controls (relaxation or psychotherapy) (SMD = −0.71; 95% CI: −1.05 to −0.37). Meta-analyses further showed that both relaxation (SMD = −0.90; 95% CI: −1.30 to −0.51), and mindfulness interventions (SMD = −0.62; 95% CI: −1.12 to −0.12), compared with non-active control, decreased fatigue levels. The estimates of heterogeneity for the four meta-analyses varied between none and moderate.

**Conclusion:**

This study found that the use of psychological interventions for MS-related fatigue management reduced fatigue in PwMS. While psychological interventions are generally considered first-line therapy for MS-related fatigue, further studies are needed to explore the long-term effect of this therapy.

## Introduction

Multiple sclerosis (MS) is a complex, demyelinating disease of the central nervous system (1, 2). Demyelinated nerve fibers can produce altered sensations and impairments in bodily functions. As a result, people with MS (PwMS) can experience a range of symptoms including numbness, double vision, cognitive difficulties, bladder problems, paralysis, blindness, and fatigue (3).

Fatigue is commonly reported by PwMS and is often one of the first symptoms of MS. Fatigue can be defined as “a subjective lack of physical and/or mental energy that is perceived by the individual or caregiver to interfere with usual and desired activities” (4). It may affect up to 80% of PwMS (5–7) and can be severe in up to 65–70% (8, 9). The prevalence of MS-associated fatigue is greater than other MS symptoms, such as difficulty within balance, weakness, and numbness (7). While fatigue is common in a range of chronic diseases, the nature of MS-related fatigue is thought to be more profound than that experienced by either healthy people or individuals with other types of illnesses (6, 10).

Multiple sclerosis-related fatigue is described as primary or secondary fatigue. Primary fatigue results from damage to the central nervous system: neuronal dysfunction, demyelination, and inflammation. Secondary fatigue arises due to other factors such as mood disorders, lack of sleep, and medication to manage MS and related symptoms (6, 11). Since fatigue is a subjective symptom, its evaluation is difficult (12, 13). Fatigue can be measured subjectively or objectively. Subjective fatigue measurement involves the use of validated scales to investigate perceived levels of fatigue. Objective measurement of fatigue uses scales to quantify the severity and impact of fatigue on daily physical, cognitive, and psychosocial activities through various parameters (6, 14–16).

Multiple sclerosis-associated fatigue adversely affects functioning, activities of daily living, employment, socialization, and quality of life (6, 17). For PwMS, fatigue may contribute to early retirement, reduced working hours, and unemployment (17–21). The level of unemployment in PwMS has been shown to be as high as 80% (22). Consequently, fatigue and associated-unemployment has significant socioeconomic implications for PwMS and their families (6, 17, 19).

There are two forms of MS-related fatigue management: non-pharmacological approaches and pharmacological management. It has been argued that non-pharmacological interventions should be considered first-line treatment (6). These can include physical, psychological and cognitive, and mixed interventions (23, 24). Physical approaches include aerobic exercises, resistance training, electromagnetic-field therapy, and cooling therapy. Psychological approaches include cognitive behavioral therapy (CBT), relaxation therapy, psychotherapy, energy conservation education, progressive muscle relaxation, mindfulness, and educational counseling (23).

Several published articles indicate that non-pharmacological interventions are effective in improving fatigue of PwMS (23, 25–30). There are comprehensive systematic reviews (25, 31, 32) in the MS literature demonstrating the effectiveness of pharmacological interventions and physical training on fatigue. Several studies have demonstrated that psychological treatments such as CBT, mindfulness, relaxation, and educational counseling, decreased the fatigue level of PwMS (33–35). However, to date, there is only one systematic review (36) exploring effects of psychological interventions. It focused exclusively on the efficacy of CBT for MS-related fatigue and it did not provide an assessment of the effects of all psychological interventions on fatigue in PwMS. Therefore, there is a need for a broader approach to investigate the efficacy of other types of psychological interventions such as mindfulness and relaxation on MS-associated fatigue management. A broad and more comprehensive systematic review concerning psychological interventions may assist in the development of clinical and research recommendations for psychological approaches for fatigue management. Our aim of this study was to determine the efficacy of psychological interventions in improving fatigue in PwMS.

## Methods

To determine the efficaciousness of psychological intervention in managing fatigue in PwMS, we defined terms of interest as follow: (a) the population of interest was PwMS who were aged 18 years or older; (b) interventions were psychological interventions; (c) comparators were non-active/active controls; (d) the outcome was fatigue; and (e) study designs included all types of studies except reviews, case reports, case series, and qualitative studies.

### Search Methods

This systematic review and meta-analysis was conducted in accordance with the preferred reporting items for systematic reviews and meta-analyses statement (37). The review is registered with PROSPERO, registration number-CRD42017060497. The following electronic bibliographic databases were searched for articles published from database inception to April 5, 2017: Medline (Ovid), EMBASE, PsycINFO, and CINAHL. We consulted with a professional librarian for assistance with search strings. Search terms included those related to MS, fatigue, and psychological interventions (Table A1 in Appendix). As preliminary searches indicated few papers published in this area, we made no restrictions on language, year of publication, or publication type in our search. In addition, we also used Web of Science to search publications which cited our included studies and we undertook a hand-search of the reference lists of a relevant systematic review (36).

### Inclusion Criteria

Articles were included if they: (a) included participants with MS who were aged 18 years or older; (b) included participants who had self-reported neurologist-diagnosed MS, or doctor-diagnosed MS, or recruitment of PwMS from MS society, clinic, and hospital; (c) assessed interventions involving psychological therapy, CBT (including self-management), stress reduction techniques, meditation, mindfulness, relaxation, guided imagery, progressive muscle relaxation, or educational counseling; (d) had a comparison group (baseline (within group) or standard-care or non-active/active-control group) or single psychological intervention group; (e) included an outcome measure for fatigue assessed using a validated tool; (f) were written in English; and (g) were full text article. In addition, we deviated from our original protocol and made *a posteriori* decision to include pilot studies in this review given that small studies can contribute meaningful information to meta-analyses.

We excluded papers not written in English, and studies with multi-component interventions that did not isolate the psychological therapy in design or analysis, literature reviews (including systematic reviews and meta-analyses), case reports, case series, or reported qualitative findings only. We contacted primary authors and co-authors when the methods described did not enable us to determine whether the inclusion criteria were met.

### Study Selection

All abstracts identified through the search were independently screened for eligibility by title and abstract by two authors (Aung Zaw Zaw Phyo and Thibaut Demaneuf). All relevant full text articles were evaluated for eligibility against inclusion criteria. Two authors independently completed the eligibility assessments. This was followed by a consensus round where disagreements between the two authors (Aung Zaw Zaw Phyo and Thibaut Demaneuf) were resolved by further consensus with authors Tracey J. Weiland and Alysha M. De Livera.

### Data Extraction

The following information was extracted independently by two authors (Aung Zaw Zaw Phyo and Thibaut Demaneuf): primary author (year of publication); country where the study took place; study design; participant characteristics (i.e., age and sex of the participants); interventions assessed; scales used to assess the outcomes; findings; and study limitations. We recorded the outcome data (i.e., mean and SD) at baseline (pre-test); end of intervention; and follow-up assessments as reported in the studies.

### Quality Appraisal

Two authors (Aung Zaw Zaw Phyo and Thibaut Demaneuf) evaluated the quality of included studies using the Effective Public Health Practice Project (EPHPP) Quality Assessment Tool for Quantitative Studies (Hamilton Tool) (38). The EPHPP evaluates six domains: (a) selection bias; (b) study design; (c) confounders; (d) blinding; (e) data collection method; and (f) withdrawals/dropouts. The EPHPP guidelines recommend that each domain is rated as strong; moderate; or weak (38). To ascertain a global rating for each study, we gave a rating of strong when there were no weak ratings across the six domains; a rating of moderate if there was one weak rating across the six domains; and a rating of weak when an article had two or more weak ratings across the six domains (38). Disagreements between reviewers were resolved through discussion and consensus with authors Tracey J. Weiland and Alysha M. De Livera. This step was not used to exclude papers from this review.

### Data Analysis

In this review, all 20 included studies were presented in a narrative synthesis. Table A2 in Appendix outlines reasons why studies were not included in this systematic review. Twelve studies (13 articles) (35, 39–50) with sufficient data were included in our meta-analyses. Table A3 in Appendix outlines reasons why the remaining eight studies (nine articles) (33, 34, 51–57) were not included in the meta-analyses.

We used post-treatment means and SDs to calculate standardized mean differences (SMDs) and 95% confidence intervals (CIs). A random-effects model was fitted using the DerSimonian and Laird estimator for the between-study variance (58). In this analysis, a negative SMD implies that fatigue is reduced in the intervention groups compared with the active/non-active control groups. According to Cohen’s definition (59), we interpreted SMDs to have a small effect if the magnitude was 0.2–0.5; moderate effect if the magnitude was 0.51–0.8, and large effect if the magnitude was >0.8. We used the *I*^2^ statistic to assess statistical heterogeneity and we interpreted these results according to the Cochrane guidelines (60) (0–40% = no heterogeneity; 30–60% = moderate heterogeneity; 50–90% = substantial heterogeneity; and 75–100% = considerable heterogeneity). Publication bias was assessed using funnel plots and Egger’s test (61).

We conducted four meta-analyses: a comparison of CBT and non-active controls; a comparison of CBT and active controls [e.g., relaxation therapy or supportive-expressive group psychotherapy (SEGP)]; a comparison of relaxation and non-active controls; and a comparison of mindfulness interventions and non-active controls. Non-active controls included waitlist, current local practice that included general advice and information provision about MS-fatigue from a variety of health professionals, standard care or treatment as usual in which the participants did not receive any psychological interventions. Data analysis was performed using Stata statistical software, version 13.0 (StataCorpLP, College Station, TX, USA).

## Results

### Search Result

**Figure 1:**
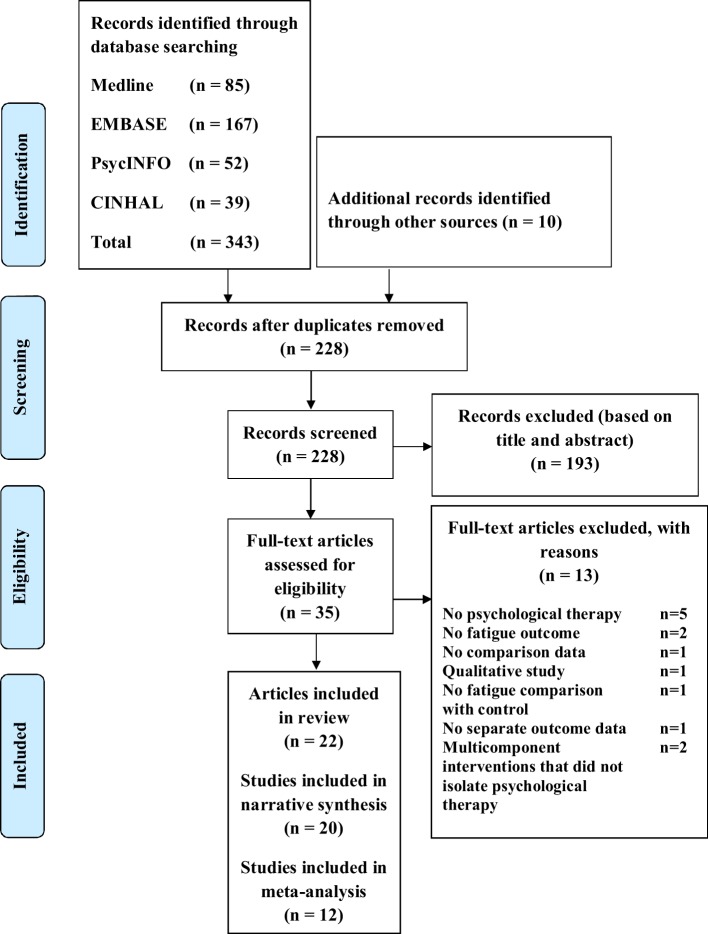
Flow diagram of review process.

We identified 343 articles from the database search and an additional 10 articles were identified from other sources (Figure 1). Of these, 228 remained for review after removal of duplicates. In total, 193 articles were deemed irrelevant based on the title and abstract. We assessed the full-text of the remaining 35 articles for eligibility. 22 articles met all inclusion criteria and were selected for this systematic review (Figure 1). Two published articles by Thomas et al. (47, 48) which reported the results of the same randomized controlled trial (RCT) were combined as one study. Similarly, two published articles by Jongen et al. (56, 57) on the same observational study were combined as one study. In total, there were 20 studies of which 1,249 PwMS were included. Excluded studies with the main reasons for exclusion are shown in Table A2 in Appendix. The quality of studies ranged from weak to strong; 10 studies (40–46, 49, 52, 53), 4 studies (35, 47, 48, 50, 55), and 6 studies (33, 34, 39, 51, 54, 56, 57) had a global rating of strong, moderate, and weak, respectively (Table 1).

**Table 1:** Quality of evidence rating for included studies based on the Effective Public Health Practice Project Quality Assessment Tool for Quantitative Studies.

Reference	Selection bias	Study design	Confounders	Blinding	Data collection method	Withdrawals and dropouts	Global rating
Alisaleh and Shahrbanoo (39)	Moderate	Strong	Weak	Moderate	Strong	Weak	Weak
Anderson et al. (51)	Moderate	Weak	Weak	Moderate	Strong	Strong	Weak
Bogosian et al. (40)	Strong	Strong	Strong	Moderate	Strong	Moderate	Strong
Carletto et al. (52)	Strong	Strong	Strong	Moderate	Strong	Strong	Strong
Dayapoglu and Tan (33)	Moderate	Moderate	Weak	Moderate	Strong	Weak	Weak
Ehde et al. (41)	Strong	Strong	Strong	Moderate	Strong	Strong	Strong
Fischer et al. (42)	Strong	Strong	Strong	Moderate	Strong	Moderate	Strong
Grossman et al. (53)	Strong	Strong	Strong	Moderate	Strong	Strong	Strong
Jongen et al. (56, 57)	Moderate	Weak	Weak	Moderate	Strong	Strong	Weak
Kiropoulos et al. (43)	Strong	Strong	Strong	Moderate	Strong	Strong	Strong
Kos et al. (44)	Moderate	Strong	Strong	Moderate	Strong	Strong	Strong
Mackay et al. (34)	Weak	Strong	Strong	Weak	Strong	Weak	Weak
Mohr et al. (45)	Strong	Strong	Strong	Moderate	Strong	Strong	Strong
Moss-Morris et al. (46)	Moderate	Strong	Strong	Moderate	Strong	Strong	Strong
Nazari et al. (35)	Moderate	Strong	Strong	Moderate	Strong	Weak	Moderate
Spitzer and Pakenham (54)	Moderate	Weak	Weak	Moderate	Strong	Strong	Weak
Thomas et al. (47, 48)	Weak	Strong	Strong	Moderate	Strong	Strong	Moderate
van Kessel et al. (49)	Strong	Strong	Strong	Moderate	Strong	Strong	Strong
van Kessel et al. (55)	Moderate	Strong	Strong	Weak	Strong	Moderate	Moderate
Vazirinejad et al. (50)	Moderate	Strong	Strong	Moderate	Strong	Weak	Moderate

### Description of Studies

The included studies were published between 2003 and 2017 and reported 11 RCTs (34, 35, 41–45, 47–50, 52, 53), 4 pilot RCTs (40, 43, 46, 55), 1 experimental and control groups pre-test/post-test pilot study (39), 1 observational study (56, 57), 1 single group pre-test and post-test pilot study (54), and 2 single-group pre-test and post-test studies (33, 51). The countries where the included studies took place were Turkey (33), the United States of America (41, 45), Germany (42), Switzerland (53), Belgium (44), Australia (34, 43, 54), Italy (52), Iran (35, 39, 50), United Kingdom (40, 46–48, 51), Netherlands (56, 57), and New Zealand (49, 55).

Of the 16 included intervention and control studies, 10 studies (35, 39–43, 46–48, 50, 53) compared psychological interventions against non-active controls and 6 studies (34, 44, 45, 49, 52, 55) compared psychological interventions against active controls. In four studies, two psychological interventions of interest were compared: CBT and supportive-expressive group (psychotherapy) (45); self-management occupational therapy (CBT) and relaxation therapy (44); and CBT and relaxation (49); and eye movement desensitization and reprocessing (EMDR) and relaxation therapy (52); respectively. Table 2 presents a summary of the 20 included studies, and Table 3 displays the characteristics of participants of each included study. Table 4 shows the proportion of the study sample that completed the follow-up assessment.

**Table 2:** Summary of data extraction from 20 included studies.

Reference	Country	Study design	Total participants	Interventions	Data collection methods	Scale used	Results (data); mean (SD)	Main findings
Alisaleh and Shahrbanoo (39)	Iran	Pre-test/post-test pilot study	In total, 30 people with MS (PwMS) Mindfulness-based stress reduction (MBSR) *n* = 15 Waiting list control *n* = 15	MBSR intervention 8 sessions (2 h per session) Waiting list control (no intervention was done)	Pre-test and post-test (after intervention)	Fatigue Severity Scale (FSS)	*Intervention* Pre-test 4.79 (1.07) Post-test 3.53 (0.80) *Control* Pre-test 4.41 (0.95) Post-test 4.23 (1.01)	Significant difference in fatigue was found between intervention and control group after intervention

Anderson et al. (51)	United Kingdom	Single group pre-test/post-test feasibility study	In total, 21 PwMS	Self-management intervention (combining positive psychology theory and practice, and CBT) for 6 weeks	Pre-test and post-test (6 weeks)	FSS	*Intervention* Baseline 49.4 (13.3) Post-test 41.4 (14.4)	Intervention decreased fatigue severity

Bogosian et al. (40)	United Kingdom	Pilot randomized controlled trial (RCT)	In total, 40 PwMS Mindfulness *n* = 19 Wait list control *n* = 21 Intention to treat analysis was conducted	Mindfulness intervention for 8 weeks Wait list control received a mix of clinical input and review of care providers (few patients routinely received treatment for distress)	Baseline Post-intervention and 3-month follow-up	FSS	*Intervention* Baseline 39.91 (14.45) Post-test 43.98 (14.20) 3-month follow-up 43.87 (13.39) *Control* Baseline 48.29 (12.24) Post-test 49.98 (10.18) 3-month follow-up 49.08 (12.43)	After intervention, mean of FSS was reduced in the intervention group compared with controls

Carletto et al. (52)	Italy	RCT	Fifty patients were randomly assigned. However, in total, 42 PwMS were included Eye movement desensitization and reprocessing treatment *n* = 20 Relaxation therapy *n* = 22	Eye movement desensitization and reprocessing (EMDR) treatment for 10 individual 60-min-long treatment sessions over 12–15 weeks Relaxation therapy for 10 individual 60-min-long treatment sessions over 12–15 weeks	Pre-treatment and post-treatment	FSS	*EMDR* Pre-test 43.10 (15.10) Post-test 37.60 (19.67) *Relaxation intervention* Pre-test 43.95 (13.79) Post-test 39.18 (15.94)	Both EMDR and relaxation therapy were effective in reducing fatigue significantly

Dayapoglu and Tan (33)	Turkey	Single-group pre-test/post-test pre-trial model	In total, 32 PwMS	Application of progressive muscle relaxation technique: once a day for 6 weeks	Pre-test and post-test (6 weeks after the completion of their education)	FSS	*Intervention* Baseline 5.75 (0.95) Post-test 3.81 (1.30)	Intervention decreased patients’ fatigue level and the difference between pre- and post-intervention was statistically significant

Ehde et al. (41)	United States of America	RCT	In total, 163 PwMS Telephone-delivered self-management telephone-delivered self-management intervention (T-SM) *n* = 75 Telephone-delivered parallel education (T-ED) *n* = 88 Intention to treat analysis was conducted	T-SM: consisted of cognitive behavioral and positive psychological strategies T-SM intervention = 8 weekly 45–60 min telephone sessions and 15 min follow-up calls at 4 weeks post-intervention and 8 weeks post-intervention T-ED—standard care control	Baseline Post-test (9–11 weeks) follow-up (6-month) Follow-up (12-month)	Modified Fatigue Impact Scale (MFIS)	*T-SM (intervention)* Baseline 48.0 (14.7) Post-test 38.6 (15.9) 6-month follow-up 37.3 (16.0) 12-month follow-up 40.2 (16.5) *T-ED (control)* Baseline 51.2 (12.7) Post-test 42.4 (15.8) 6-month follow-up 41.7 (16.2) 12-month follow-up 43.3 (15.8)	In both groups, there was significant improvement in fatigue outcome from baseline to post-intervention

Fischer et al. (42)	Germany	RCT	In total, 90 PwMS Deprexis *n* = 45 patients Control group *n* = 45 patients Intention-to-treat analysis was used	Intervention group (Deprexis—Internet-based CBT) for 9 weeks Controls-Waitlist Control Group	Baseline Post-treatment Follow-up—6 months after the end of the intervention	Fatigue Scale for Motor and Cognitive Function; (FSMC)	*Intervention* (FSMC Total) Baseline 74.18 (13.51) Post-test 70.15 (16.06) *Control* (FSMC Total) Baseline 71.84 (16.06) Post-test 70.87 (15.22)	Intervention decreased fatigue however, statistically significant improvement was only observed for the Fatigue Scale (Motor)

Grossman et al. (53)	Switzerland	RCT	In total, 150 PwMS Mindfulness-based intervention (MBI) *n* = 76 Usual care (UC) *n* = 74 Intention-to-treat analysis was used	Intervention group received a structured 8-week program of mindfulness training UC was additionally applied to this group UC group is control group	Baseline Post-treatment 6-month follow-up	MFIS	*MBI Group (Intervention)* Baseline 35.15 (16.68) Post-test 6.65 (Mean Change) 6-month follow-up 6.58 (Mean Change) *Control* Baseline 30.28 (14.98) Post-test −0.10 (Mean Change) 6-month follow-up −0.71 (Mean Change) *Positive change indicates improvement	Compared with UC, MBI improved fatigue up to 6-month post-intervention

Jongen et al. (56, 57)	Netherlands	Observational study	57 PwMS completed baseline assessment. However, in total, 47 PwMS were included	Intensive social cognitive treatment (can do treatment) with participation of support partners for 3 days	Baseline, 1, 3, 6, and 12 months after intervention	MFIS	*Intervention* *For relapsing remitting MS people* Baseline 12.72 (3.16) 1-month 11.00 (3.31) 3-month 10.94 (3.59) 6-month 11.89 (3.55) 12-month 9.95 (3.77) *Intervention* *For progressive MS people* Baseline 12.09 (4.08) 1-month 12.19 (3.53) 3-month 11.77 (3.95) 6-month 12.05 (3.50) 12-month 11.93 (3.36)	There was no statistically significant decrease in the level of MS related fatigue

Kiropoulos et al. (43)	Australia	Pilot RCT	In total, 30 PwMS CBT *n* = 15 Treatment as usual *n* = 15	CBT (8-week tailored intervention) Treatment as usual was control and did not obtain any psychological treatment	Pre-test, post-test, and 20-week follow-up	MFIS	*Intervention* Pre-test 12.13 (3.58) Post-test 8.73 (3.58) 20-week follow-up 8.06 (3.03) *Control* Pre-test 12.26 (3.84) Post-test 11.93 (4.38) 20-week follow-up 11.06 (4.74)	CBT showed significant reductions in level of fatigue

Kos et al. (44)	Belgium	RCT	In total, 31 PwMS Self-management occupational therapy (SMOoTh Group) *n* = 17 Relaxation group *n* = 14 Intention-to-treat analysis was used.	Both groups received three individual sessions (60–90 min for 3 weeks) SMOoTh was based on guidelines of MS Council and the Energy Conservation/Envelope Theory. (Partial CBT) Relaxation Therapy including education on the role of stress management in MS and practicing relaxation techniques pending individual preferences	Baseline Post-treatment 3-month follow-up	MFIS	*SMOoTh Group* (MFIS Total) Baseline 43.5 (8.5) Post-test 33.9 (11.4) 3-month follow-up 32.3 (11.1) *Relaxation Group* (MFIS Total) Baseline 44.9 (14.3) Post-test 39.3 (13.1) 3-month follow-up 41.9 (15.4)	The impact of fatigue was decreased post-intervention compared with baseline in both groups

Mackay et al. (34)	Australia	RCT	In total, 40 PwMS Relaxation, mindfulness, social support, and education (RMSSE) plus Biofeedback *n* = 20 RMSSE *n* = 20	RMSSE Group Received one—1 h session per week for 3 weeks—about RMSSE RMSSE plus Biofeedback Group Received RMSSE and additionally received biofeedback	Baseline End of treatment At follow-up (3 months after the last appointment)	FSS	*RMSSE Group* Baseline 4.91 (1.60) Post-test 4.38 (1.90) *RMSSE plus* *Biofeedback* Baseline 4.96 (1.21) Post-test 3.96 (1.47)	No significant pre-and post-treatment improvement in FSS (RMSSE Group) Significant pre-and post-treatment improvement in FSS (RMSSE plus Biofeedback Group)

Mohr et al. (45)	United States of America	Randomized clinical trial	In total, 60 PwMS CBT *n* = 22 Supportive-expressive group; psychotherapy (SEGP) *n* = 22 Sertraline medication *n* = 16	CBT: individual CBT consisted of 16 weekly, individual 50 min meetings with a psychologist one per week for 16 weeks SEGP: groups of 5–9 patients and two psychologists met for 16 weekly 90-min sessions focused on enhancing emotional expression and the social and personal sequelae of the disease Sertraline: an antidepressant medication	Baseline End of treatment (at week 16)	Fatigue severity [global fatigue severity (GFS)] subscale of the fatigue assessment instruments (FAI)	*CBT* Baseline 58.2 (8.67) Post-test 52.5 (12.52) *SEGP* Baseline 60.7 (8.83) Post-test 61.3 (9.89)	Scores on the global fatigue severity subscale were significantly reduced over the course of interventions

Moss-Morris et al. (46)	United Kingdom	Pilot RCT	In total, 40 PwMS 23 participants in Web Based Self-management (CBT) Msinvigor8 17 participants as Standard care controls	Web Based Self-management (CBT) Msinvigor8 consisted of 8 weekly sessions (on average, sessions took 25–50 min). Additionally, participants received three telephone support session of between 30 and 60 min Standard care group was control group	Pre-test and Post-test (10 weeks)	Fatigue scale (FS) and MFIS	*Intervention* *Baseline* FS 21.39 (4.30) MFIS 13.17 (3.81) *Post-test* FS 12.39 (6.84) MFIS 9.00 (3.75) *Control* *Baseline* FS 21.53 (3.62) MFIS 12.69 (3.89) *Post-test* FS 19.57 (5.20) MFIS 12.88 (3.89)	Intervention significantly decreased MS-related fatigue

Nazari et al. (35)	Iran	RCT	In total, 75 PwMS 25 patients in Reflexology 25 patients in Relaxation 25 patients in control group	Intervention of relaxation was performed for 4 weeks (twice a week for 40 min in each session) The control group received only routine treatment and care recommended by the attending physician	Before Immediately after intervention 2 months after intervention	FSS	*Relaxation Group* Baseline 4.93 (0.87) Post-test 4.12 (0.83) 2-month follow-up 4.37 (0.78) *Control Group* Baseline 4.89 (0.95) Post-test 4.78 (1.01) 2-month follow-up 4.74 (0.86)	There was a significant difference between the two groups of relaxation and control immediately after-follow up

Spitzer and Pakenham (54)	Australia	Mixed-methods pilot study (single group, pre- and post-intervention)	In total, 23 PwMS Intention to treat analysis was conducted	Mindfulness programs for 5 weekly sessions (2 h per session)	Pre-intervention Post-intervention 8-week follow-up	MFIS	*Intervention* Baseline 2.32 (0.90) Post-test 2.17 (0.73) 8-week follow-up 2.33 (0.77)	Intervention did not alter levels of fatigue

Thomas et al. (47, 48)	United Kingdom	RCT	In total, 164 PwMS FACETS group *n* = 84 CLP group *n* = 80 *Fatigue: applying Cognitive behavioral and Energy effective Techniques to lifestyle (FACETS) *Current Local Practice alone (CLP)	Intervention—FACETS consisted of cognitive behavioral, social-cognitive, energy effectiveness, self-management, and self-efficacy theories Intervention included six sessions (90 min duration) held weekly and facilitated in groups of 6–12 by two health professionals. Intervention group is FACETS plus current local practice CLP (control) is current local practice which consisted of general advice and information provision (advice) from health professionals	Base (1 week before FACETS) 1-month (follow-up 1) 4-month (follow-up 2) 12-month (follow-up 3) after final session	Fatigue severity (GFS) subscale of the FAI	*FACETS Group (Intervention)* Baseline 5.60 (0.98) 1-month follow-up 5.48 (0.92) 4-month follow-up 5.26 (1.03) 12-month follow-up 5.32 (1.00) *CLP Group* Baseline 5.61 (1.09) 1-month follow-up 5.55 (1.17) 4-month follow-up 5.66 (0.93) 12-month follow-up 5.70 (1.01)	FACETS intervention is effective in decreasing fatigue in PwMS

van Kessel et al. (49)	New Zealand	RCT	In total, 72 PwMS people Cognitive Behavioral Therapy (CBT) *n* = 35 Relaxation training (RT) *n* = 37 Intention-to-treat analysis was used	Eight weekly sessions of CBT Eight weekly sessions of RT	Pre-treatment Post-treatment (2 month) 3-month follow-up 6-month follow-up	Fatigue scale	*CBT* Baseline 20.94 (4.25) Post-test 7.90 (4.34) 3-month follow-up 8.99 (5.31) 6-month follow-up 10.37 (6.37) *RT* Baseline 20.32 (4.28) Post-test 11.57 (5.28) 3-month follow-up 11.11 (4.57) 6-month follow-up 12.49 (5.24)	Both CBT and RT groups were effective in decreasing fatigue and the decrease was clinically significant

van Kessel et al. (55)	New Zealand	Pilot RCT	In total, 39 PwMS MSInvigor8-Only group *n* = 20 MSInvigor8-Plus *n* = 19	MSInvigor8 was an eight-session Internet-delivered treatment program based on CBT protocol (each session took between 25 and 50 min) MSInvigor8-Only was the MSInvigor8 program without any therapeutic contact. MSInvigor8_Plus was the MSInvigor8 program with email support from a clinical psychologist	Pre-test and Post-treatment (10 weeks)	Fatigue scale (FS) and MFIS	*MSInvigor8 Only Group* *Pre-test* FS 20.80 (3.12) MFIS 13.85 (2.98) *Post-test* FS 17.50 (6.37) MFIS 12.35 (3.65) *MSInvigor8-Plus* *Pre-test* FS 22.37 (4.39) MFIS 13.58 (2.97) *Post-test* FS 11.37 (6.20) MFIS 10.00 (2.71)	MSInvigor8-Plus intervention showed significant greater reduction in fatigue severity and fatigue impact compared with the MSInvigor8-Only group

Vazirinejad et al. (50)	Iran	RCT	In total, 60 PwMS Psychological training with gradual muscle relaxation *n* = 30 Control *n* = 30	Psychological training with gradual muscle relaxation (12 sessions, 2 sessions per week) Control is no intervention (no training, no muscle relaxation and no other treatment)	Baseline Immediately after an intervention Three months after an intervention	FSS	*Intervention Group* Baseline 42.83 (8.36) Post-test 33.90 (7.07) 3-month follow-up 35.57 (7.61) *Control* Baseline 41.90 (6.67) Post-test 41.00 (6.10) 3-month follow-up 41.10 (5.57)	A significant reduction in the FSS was found in the education group

**Table 3:** Summary of characteristics of participants of each included studies.

Reference	Characteristics of participants *n*(%)	
Alisaleh and Shahrbanoo (39)	*Mindfulness-based Stress Reduction and Waiting List Control n = 30* *Age* Range = 20–40	

Anderson et al. (51)	*Self-management Intervention n = 21* *Age* Mean (SD) = 54.3 (10.5) Range = 36–76 *Gender* Male = 4 (19) Female = 17 (81) *Type of MS* Relapsing–remitting = 12 (57.1) Secondary progressive = 8 (38.1) Benign = 1 (4.8)	

Bogosian et al. (40)	*Mindfulness n = 19* *Age* Mean (SD) = 53.42 (8.3) *Gender* Female = 9 (47.4) *Type of MS* Primary progressive = 5 (26.3) *EDSS* Mean (SD) = 6.8 (1.6)	*Waiting List Control n = 21* *Age* Mean (SD) = 50.9 (9.9) *Gender* Female = 13 (61.9) *Type of MS* Primary progressive = 12 (57.1) *EDSS* Mean (SD) = 6.2 (1.4)

Carletto et al. (52)	*Eye Movement Desensitization and Reprocessingn = 20* *Age* Mean (SD) = 39.52 (11.68) *Gender* Male = 5 (25) Female = 15 (75) *Type of MS* Relapsing–remitting = 17 (85) Primary progressive = 1 (5) Secondary progressive = 2 (10) *EDSS* Mean (SD) = 2.00 (4.50)	*Relaxation Therapy Group n = 22* *Age* Mean (SD) = 40.66 (10.03) *Gender* Male = 3 (13.64) Female = 19 (86.36) *Type of MS* Relapsing–remitting = 19 (86.36) Primary progressive = 1 (4.54) Secondary progressive = 2 (9.1) *EDSS* Mean (SD) = 2.00 (1.60)

Dayapoglu and Tan (33)	*Progressive Muscle Relaxation n = 32* *Age* 24–33 = 12 (37.5) 34–43 = 11 (34.4) 44–53 = 5 (15.6) 54 and above = 4 (12.5) *Gender* Male = 12 (37.5) Female = 20 (62.5)	

Ehde et al. (41)	*Self-management Intervention n = 75* *Age* Mean (SD) = 51.0 (10.1) Range = 25–75 *Gender* Male = 8 (10.7) Female = 67 (89.3) *Type of MS* Relapsing–remitting = 46 (61.3) Progressive = 29 (38.7) *EDSS* ≤4.0 = 19 (25.3) 4.5–6.5 = 46 (61.3) ≥7.0 = 10 (13.3)	*Parallel Education Intervention n = 88* *Age* Mean (SD) = 53.2 (10.0) Range = 26–76 *Gender* Male = 13 (14.8) Female = 75 (85.2) *Type of MS* Relapsing–remitting = 45 (51.1) Progressive = 43 (48.9) *EDSS* ≤4.0 = 23 (26.1) 4.5–6.5 = 55 (62.5) ≥7.0 = 10 (11.4)

Fischer et al. (42)	*Deprexis—Internet-based CBT n = 45* *Age* Mean (SD) = 45.36 (12.64) *Gender* Male = 11 (24) Female = 34 (76) *Type of MS* Clinically isolated syndrome = 3 (7) Relapsing–remitting = 21 (47) Primary progressive = 7 (16) Secondary progressive = 9 (20) Unclear = 5 (11)	*Wait List Control n = 45* *Age* Mean (SD) = 45.20 (10.56) *Gender* Male = 9 (20) Female = 36 (80) *Type of MS* Clinically isolated syndrome = 3 (7) Relapsing–remitting = 19 (42) Primary progressive = 4 (9) Secondary progressive = 12 (27) Unclear = 7 (16)

Grossman et al. (53)	*Mindfulness n = 76* *Age* Mean (SD) = 45.93 (10.00) *Gender* Female = 59 (78) *Type of MS* Relapsing–remitting = 60 (79) *EDSS* Mean (SD) = 3.03 (1.12)	*UC n = 74* *Age* Mean (SD) = 48.68 (10.58) *Gender* Female = 60 (81) *Type of MS* Relapsing–remitting = 63 (85) *EDSS* Mean (SD) = 2.98 (0.83)

Jongen et al. (56, 57)	*Intensive Social Cognitive Treatment n = 47* *Types of MS* Relapsing–remitting = 20 Progressive = 24 *For* relapsing–r*emitting group n = 20* *Age* Mean (SD) = 42.7 (10.1) Range = 25–65 *Gender* Male = 4 (20) Female = 16 (80) *EDSS* Mean (SD) = 3.1 (1.2) *For Progressive group n = 24* *Age* Mean (SD) = 48.7 (7.6) Range = 30–60 *Gender* Male = 5 (20.83) Female = 19 (79.17) *EDSS* Mean (SD) = 5.5 (1.4)	

Kiropoulos et al. (43)	*CBT n = 15* *Age* Mean (SD) = 34.60 (9.06) *Gender* Female = 13 (86.7) *Type of MS* Relapsing–remitting = 15 (100)	*Treatment as Usual n = 15* *Age* Mean (SD) = 39.27 (9.93) *Gender* Female = 9 (60) *Type of MS* Relapsing–remitting = 15 (100)

Kos et al. (44)	*Self-management Occupation Therapy n = 17* *Age* Mean (SD) = 37 (8.2) *EDSS* Median (IQR) = 3 (2.5–3.25)	*Relaxation n = 14* *Age* Mean (SD) = 44 (8.9) *EDSS* Median (IQR) = 3.5 (3.5–4)

Mackay et al. (34)	*Relaxation, Mindfulness, Social Support and Education and Biofeedback n = 20* *Age* Mean (SD) = 45.45 (13.34) *Gender* Male = 5 (25) Female 15 (75) *Type of MS* Relapsing–remitting = 20 (100) *EDSS* Mean (SD) = 2.41 (1.84)	*Relaxation, Mindfulness, Social Support and Education n = 20* *Age* Mean (SD) = 46.35 (11.76) *Gender* Male = 3 (15) Female = 17 (85) *Type of MS* Relapsing–remitting = 20 (100) *EDSS* Mean (SD) = 2.41 (1.57)

Mohr et al. (45)	*CBT, Psychotherapy and Sertraline medication n = 60* *Age* Mean (SD) = 44.6 (10.3) *Gender* Female = 43 (71.7) *Type of MS* Relapsing form = 60 (100)	

Moss-Morris et al. (46)	*CBT Msinvigor8 n = 23* *Age* Mean (SD) = 40.14 (17.76) *Gender* Female = 16 (69.6) *Type of MS* Relapsing–remitting = 10 (43.5) Primary progressive = 2 (8.7) Secondary progressive = 7 (30.4) Unsure = 4 (17.4)	*Standard Care n = 17* *Age* Mean (SD) = 41.81 (11.43) *Gender* Female = 16 (94.1) *Type of MS* Relapsing–remitting = 12 (70.6) Primary progressive = 0 (0) Secondary progressive = 2 (11.8) Unsure = 3 (17.6)

Nazari et al. (35)	*Relaxation n = 25* *Age* Mean (SD) = 33.90 (5.60) *Gender* Female = 25 (100)	*Control n = 25* *Age* Mean (SD) = 34.40 (7.70) *Gender* Female = 25 (100)

Spitzer and Pakenham (54)	*Mindfulness n = 23* *Age* Mean (SD) = 48.4 (9.6) Range = 33.0–71.6 *Gender* Male = 2 (8.7) Female = 21 (91.3) *Type of MS* Relapsing–remitting = 18 (78.3) Primary progressive = 3 (13) Secondary progressive = 2 (8.7)	

Thomas et al. (47, 48)	*FACETS CBT n = 84* *Age* Mean (SD) = 48.0 (10.2) Range = 23–73 *Gender* Male = 23 (27) Female = 61 (73) *Type of MS* Benign = 4 (5) Relapsing–remitting = 35 (43) Primary progressive = 5 (6) Secondary progressive = 16 (20) Don’t know = 21 (26) Not Stated = 3	*Current Local Practice n = 80* *Age* Mean (SD) = 50.1 (9.1) Range = 28–70 *Gender* Male = 22 (28) Female = 58 (73) *Type of MS* Benign = 2 (3) Relapsing–remitting = 40 (51) Primary progressive = 8 (10) Secondary progressive = 23 (29) Don’t know = 5 (16) Not Stated = 2

van Kessel et al. (49)	*CBT n = 35* *Age* Mean (SD) = 42.89 (9.29) *Gender* Female = 28 (80) *Types of MS* Relapsing–remitting = 23 (65.7) Primary progressive = 1 (2.9) Secondary progressive = 11 (31.4) *EDSS* Mean (SD) = 3.04 (1.78)	*RT n = 37* *Age* Mean (SD) = 47.03 (9.45) *Gender* Female = 27 (70) *Types of MS* Relapsing–remitting = 18 (48.7) Primary progressive = 8 (21.6) Secondary progressive = 11 (29.7) *EDSS* Mean (SD) = 3.86 (1.53)

van Kessel et al. (55)	*MSInvigor8-Plus n = 19* *Age* Mean (SD) = 42.95 (8.16) *Gender* Female = 11 (58.0) *Types of MS* Relapsing–remitting = 15 (79.0) Secondary progressive = 2 (10.5) Not known = 2 (10.5) *EDSS* 0–4 = 10 (53.0) 4.5–5.5 = 2 (10.5) 6–6.5 = 6 (31.5) Not recorded = 1 (5.0)	*MSInvigor8-Only n = 20* *Age* Mean (SD) = 45.70 (8.39) *Gender* Female = 18 (90.0) *Types of MS* Relapsing–remitting = 11 (55.0) Secondary progressive = 3 (15.0) Not known = 6 (30.0) *EDSS* 0–4 = 8 (40.0) 4.5–5.5 = 7 (35.0) 6–6.5 = 3 (15.0) Not recorded = 2 (10.0)

Vazirinejad et al. (50)	*Psychological Training with Gradual Muscle Relaxation n = 30* *Age* Mean (SD) = 32.6 (6.355) *Gender* Female = 24 (80)	*Control n = 30* *Age* Mean (SD) = 31.8 (6.687) *Gender* Female = 23 (76.7)

*EDSS, expanded disability status scale*.

**Table 4:** Summary of follow-up proportions for intervention and control groups (15 studies).

Reference	Follow-up proportions of intervention group	Follow-up proportions of control group/active control–intervention group
Anderson et al. (51)	100% completed at post-intervention assessment	Nil

Bogosian et al. (40)	90% completed at post-intervention assessment 79% completed at 3-month follow-up assessment	91% completed at post-intervention assessment 86% completed at 3-month follow-up assessment

Carletto et al. (52)	80% completed at post-intervention assessment	88% completed at post-intervention assessment

Dayapoglu and Tan (33)	91% completed at post-treatment assessment	Nil

Ehde et al. (41)	85% completed at post-treatment assessment 83% completed at 6-month assessment 80% completed at 12-month assessment	92% completed at post-treatment assessment 90% completed at 6-month assessment 91% completed at 12-month assessment

Fischer et al. (42)	78% completed at follow-up	80% completed at follow-up

Grossman et al. (53)	95% completed at post-treatment assessment 93% completed at 6-month follow-up	91% completed at post-treatment assessment 91% completed at 6-month follow-up

Jongen et al. (56, 57)	77% completed at 1, 3, 6-month follow-up 67% completed at 12-month follow-up	Nil

Kiropoulos et al. (43)	100% completed at 8-week follow-up 100% completed at 20-week follow-up	100% completed at 8-week follow-up 100% completed at 20-week follow-up

Kos et al. (44)	100% completed at post-treatment assessment 82% completed at 3-month follow-up	93% completed at post-treatment assessment 79% completed at 3-month follow-up

Moss-Morris et al. (46)	87% completed at 10-week follow-up	73% completed at 10-week follow-up

Spitzer and Pakenham (54)	91% completed at post-treatment assessment 83% completed at 8-week follow-up	Nil

Thomas et al. (47, 48)	85% completed at 1-month post-treatment 85% completed at 4-month post-treatment 77% completed at 12-month post-treatment	94% completed at 1-month post-treatment 96% completed at 4-month post-treatment 90% completed at 12-month post-treatment

van Kessel et al. (49)	100% completed at post-treatment follow-up 100% completed at 3-month follow-up 97% completed at 6-month follow-up	95% completed at post-treatment follow-up 95% completed at 3-month follow-up 95% completed at 6-month follow-up

van Kessel et al. (55)	79% completed at post-treatment follow-up	45% completed at post-treatment follow-up

### Narrative Synthesis of All Included Studies

Alisaleh and Shahrbanoo (39) conducted a pre-test/post-test pilot study to examine the effectiveness of mindfulness-based stress reduction (MBSR) on stress and fatigue in PwMS. A total of 30 PwMS were randomly allocated into either the MBSR group or control group (no intervention—waiting list). The MBSR intervention consisted of eight sessions (2 h per session). The fatigue severity measurement was performed at baseline and at post-intervention by using the Fatigue Severity Scale (FSS). The mean (SD) of FSS for treatment group and control group after intervention were 3.52 (0.80) and 4.23 (1.01), respectively. The authors found that there were significant differences between the two groups in terms of fatigue severity after intervention [*P*-value < 0.001—as reported by Alisaleh and Shahrbanoo (39)] The validity of this study was weakened by the small sample size which may have limited generalizability and reliability. Furthermore, no information about the randomization process, participation rate, and blinding was provided. Hence, it is difficult to determine the quality of the study.

Anderson et al. (51) performed a single-group pre-test/post-test study to describe the development and feasibility of the help to overcome problems effectively self-management intervention for PwMS. In total, 21 PwMS received a self-management intervention which included positive psychology theory practices and CBT for 6 weeks. The severity of fatigue was measured by the FSS at baseline and after the intervention (6 weeks). The authors found a decrease in FSS mean for the intervention group [baseline: 49.4 (13.3), and post-treatment: 41.4 (14.4)]. However, the quality of these findings may have been negatively affected by some limitations such as lack of randomization, self-selected sample, and small sample size. It is possible that the self-selected sample have a natural setup to answer favorably to a positive psychological intervention for MS treatment.

Bogosian et al. (40) conducted a pilot RCT to assess the potential effectiveness of mindfulness training for progressive MS. A total of 40 PwMS were randomly assigned into two groups (mindfulness intervention *n* = 19 and waiting list control group *n* = 21). The mindfulness intervention of this study was delivered in 8 h-long sessions over an 8-week period through Skype video conferences. Fatigue was assessed using the FSS and measurements were collected at baseline, post-intervention, and 3-month follow-up. The mean (SD) FSS at three time points for the intervention and control groups were 39.91 (14.45), 43.98 (14.20), 43.87 (13.39), and 48.29 (12.24), 49.98 (10.18), and 49.08 (12.43), respectively. In this study, according to the nature of the intervention, it was not feasible to keep staff (clinical supervisors) or participants blind to treatment assignments. Hence, over-performance may have existed among participants from mindfulness intervention and a Hawthorne effect could have occurred. Moreover, the outcome measures were dependent on the participants’ self-reported data. Therefore, such weaknesses may have negatively affected the validity of the study.

Carletto et al. (52) reported a RCT to compare the efficacy of EMDR and relaxation therapy in PwMS. A total of 42 PwMS were included in this study (EMDR *n* = 20; and relaxation therapy *n* = 22). EMDR intervention was in fact the psychological intervention. Relaxation therapy consisted of a series of relaxation techniques such as diaphragmatic breathing, progressive muscle relaxation, visualization, cue-controlled relaxation, and rapid relaxation. Participants from both groups received 10 individual 60-min-long sessions for 12–15 weeks. Fatigue levels, assessed with the FSS, were measured pre-test and post-test. The mean FSS was 43.10 (15.10) at pre-test and 37.60 (19.67) at post-test for EMDR group and 43.95 (13.79) at pre-test and 39.18 (15.94) at post-test for relaxation therapy group, respectively. The authors concluded that both relaxation therapy and EMDR were significantly effective for fatigue among PwMS (*P*-value = 0.03). Although the considerable numbers of PwMS that were screened, the study was underpowered due to the small sample size. Therefore, this limitation may have affected the quality of this study.

Dayapoglu and Tan (33) conducted a single-group pre-test/post-test study in a neurology clinic to investigate the effect of Progressive Muscle Relaxation Technique (PMRT) on fatigue and sleep quality in PwMS. In total, 32 PwMS who met inclusion criteria were provided an intervention involving approximately 1 h of one-to-one patient education about PMRT, and listening to a compact disk on relaxation exercises. After the education, each patient was asked to perform the exercises at home once a day for 6 weeks. Data collection was conducted at two points: pre-intervention and post-intervention (6 weeks after the completion of education). The FSS was used for fatigue measurement. A statistically significant difference was found between the average FSS score pre- and post-intervention (*P*-value < 0.001). The validity of these results, however, may have been affected by several weaknesses including lack of randomization, participants and assessors knowing their types of intervention (no blinding), and no follow-up to ensure patients were correctly using the PMRT at home.

Ehde et al. (41) conducted a RCT to evaluate the efficacy of a telephone-delivered self-management intervention-(T-SM) for fatigue in PwMS and compared T-SM against non-active intervention (telephone-delivered parallel education—T-ED). A total of 163 patients were randomly allocated to either 8-week T-SM or T-ED. The T-SM consisted of evidence-based CBT and positive psychology strategies. Telephone outcome assessments were conducted at baseline, post-test, and at 6- and 12-month post-randomization. Fatigue was measured by using the Modified Fatigue Impact Scale (MFIS). In both groups, there were significant improvements from baseline to post-treatment in the fatigue outcome measure. (Within groups 95% CIs of T-SM and T-ED were 7.01–13.8 and 5.27–12.0, respectively.) Research assistants knowing a participant’s allocation and participants being aware of their interventions may have negatively affected the validity of the study.

Fischer et al. (42) presented a RCT to investigate the efficacy of an Internet-based CBT program, “Deprexis,” among PwMS. This study compared the “Deprexis” program against the waitlist (non-active control). In total, 90 PwMS were randomly allocated into two groups (45 participants in each group) for 9 weeks. An online questionnaire including a measure of fatigue was completed at baseline, 9 weeks after enrollment, and 6 months after the intervention. The Fatigue Scale for Motor and Cognitive Function (FSMC) was used to assess fatigue. The authors reported that the intervention decreased the FSMC-total scores from 74.18 (baseline) to 70.15 (post-treatment). The authors concluded that improvement with intervention versus control was only found in the motor fatigue subscale. Weaknesses of this study include participants not being blinded to their allocated interventions, which may have led to a Hawthorne effect. Moreover, the lack of a data monitoring committee to assess adverse events, one of the investigators knowing the treatment allocation, and small sample size posed high risks of bias and may have affected the validity of the findings.

Grossman et al. (53) conducted a RCT to examine the effects of a mindfulness-based intervention (MBI) compared with non-active control (usual care—UC) among PwMS. The MBI was a structured 8-week program of mindfulness training. A total of 150 PwMS were randomly allocated into two groups (76 patients in MBI and 74 patients in UC). All patient-reported outcome measures were administered at pre-intervention, post-intervention, and 6-month follow-up. For fatigue measurement, the MFIS was used. The direct post-intervention change and 6-month follow-up change of MBI group were 6.65 (95% CI: 4.14–9.16) and 6.58 (95% CI: 3.63–9.53), respectively. The authors concluded that these positive changes indicated that MBI was beneficial in managing MS-related fatigue and MBI compared with UC improved fatigue up to 6-month post-treatment. The significant weakness of this study was the lack of information about blinding of participants, which is important to determine the validity of the study. When the participants are aware of the alternative arm, results may be subject to contamination. Therefore, a Hawthorne effect could have occurred. Participants from the intervention arm may have over-performed, and the control arm participants may have felt increased levels of hopelessness or depression (if dissatisfied with their allocation group) resulting in greater fatigue symptom. These weaknesses may have negatively affected the validity of the study.

Jongen et al. (56, 57) conducted an observational study to examine the effect of a multidisciplinary, 3-day, social cognitive wellness program with the participation of support partners (social cognitive can do program—SCDP). SCDP was primarily a sociologically oriented approach to reduce the stressors that confine PwMS to their physical, psychological, or social roles. In total, 47 PwMS included in this program and the fatigue outcome assessments were conducted using MFIS (5-item version) at baseline, 1, 3, 6, and 12 months after the program. The authors categorized the study participants into two groups: (a) people with relapsing remitting MS and (b) people with progressive types of MS. The mean (SD) of MFIS at baseline, 1, 3, 6, and 12 months after the program were 12.72 (3.16), 11.00 (3.31), 10.94 (3.59), 11.89 (3.55), and 9.95 (3.77) for people with relapsing remitting MS, and 12.09 (4.08), 12.19 (3.53), 11.77 (3.95), 12.05 (3.50), and 11.93 (3.36) for people with progressive type of MS, respectively. The authors concluded that the intervention had no statistically significant decrease in the level of MS-related fatigue. This study was limited by its small sample size and the outcome assessment using self-report questionnaires which may have affected the quality of the study.

Kiropoulos et al. (43) reported a pilot RCT to examine the acceptability and effectiveness of an 8-week individual tailored CBT for depressive symptoms in PwMS. Thirty PwMS were randomly allocated into either CBT or the control group (treatment as usual). The 5-item MFIS was used to measure fatigue impact. The outcome assessments were measured at baseline, post-intervention, and 20-week follow-up. The mean (SD) of MFIS of both CBT and control groups were 12.13 (3.58), 8.73 (3.58), 8.06 (3.03), and 12.26 (3.84), 11.93 (4.38), 11.06 (4.74), respectively. The authors found that CBT produced a significant reduction in fatigue level among PwMS. This pilot RCT had limitations which may affected the quality of the study such as lack of blinding to treatment allocation of participants and staff; clinicians delivering the interventions also administered assessment questionnaires to participants; small sample size; and reliance on self-reported measures may affect the quality of the study.

Kos et al. (44) undertook a single-blind RCT to evaluate the effectiveness of an individual face-to-face SMOoTh intervention program versus relaxation among PwMS. Thirty-one PwMS were randomly allocated to two groups (17 patients in SMOoTh and 14 in relaxation). Both interventions consisted of three individual sessions of 60–90 min for three consecutive weeks. While SMOoTh included the component of partial-CBT, relaxation involved education on the role of stress management and practicing relaxation techniques. By using questionnaires, a researcher blinded to participants’ treatment allocation performed assessments at baseline, post-intervention, and 3-month follow-up. MFIS was used for fatigue measurement. According to the findings, the means of SMOoTh and relaxation groups were 43.5 (8.5) and 44.9 (14.3), respectively, at baseline. Both interventions decreased fatigue levels and the means (SD) were 33.9 (11.4) and 39.3 (13.1), respectively, at post-intervention. This study was weakened by the small sample size which may have limited generalizability and reliability. No information about participation rate was provided, and patients were aware of their assigned interventions. This may have increased the risk of bias affecting the validity of the results.

Mackay et al. (34) conducted a RCT in three sites in Sydney, Australia to investigate the effect of biofeedback in PwMS. It consisted of two interventions: (a) relaxation, mindfulness, social support, and education (RMSSE) intervention and (b) RMSSE plus biofeedback. Forty participants (20 per group) were randomly assigned to either the RMSSE or RMSSE plus biofeedback group for 3 weeks. Assessments were conducted at baseline, post-treatment, and 3-month follow-up. The FSS was used for fatigue measurement. The authors found that at post-treatment, the FSS mean scores for each of the groups were 3.96 (PMSSE-plus-biofeedback) and 4.38 (RMSSE). The biofeedback group revealed significant pre- to post-treatment improvement in fatigue (1.00; 95% CI 0.14–1.86; *P*-value = 0.02). The weaknesses of this study were small sample size, less than half of the cohort responding to the 3-month follow-up, participants in the biofeedback group knew their intervention status, less than 60% of selected individuals agreed to participate, and lack of blinding to assessors. These limitations affect the quality of the study and may have led to decreased validity of estimates.

Mohr et al. (45) performed a clinical trial to investigate the effects of treatment on fatigue in PwMS. Sixty patients with a relapsing form of MS and moderate-to-severe depression were randomly allocated to one of three validated 16-week treatments for depression: (a) individual CBT; (b) SEGP; (c) sertraline: antidepressant medication. Outcome assessments were undertaken before and after treatment. The Global Fatigue Severity subscale of Fatigue Assessment Instrument (FAI) was used for fatigue measurement. According to the findings, the mean FAI score in the CBT and SEGP groups were 58.2 (8.67) and 60.7 (8.83), respectively, at baseline. Post-intervention both groups had decreased fatigue levels and the means (SD) were 52.5 (12.52) and 61.3 (9.89), respectively. The authors found fatigue severity were significantly reduced over the course of depression treatment [*P*-value < 0.02 as reported by Mohr et al. (45)]. Lack of information about follow-up and the fact that the treatment assignment strategy was not purely random were the limitation of this study which may have affected the validity of this study.

Moss-Morris et al. (46) conducted a pilot RCT of an Internet-based CBT self-management for fatigue treatment in PwMS. A total of 40 PwMS were included in this study (Web-based CBT self-management MSInvigor8 *n* = 23 and standard care controls *n* = 17). The MSInvigor8 (web-based CBT) consisted of eight weekly sessions each taking between 25 and 50 min on average. Fatigue severity was measured using the original version of the Fatigue Scale and fatigue impact was assessed by the MFIS. The outcome assessments were taken at baseline and post-test (10 weeks). The authors found the web-based CBT intervention decreased MS-related fatigue and the mean (SD) fatigue score for both the web-based CBT group and control group were 21.39 (4.30), 12.39 (6.84), and 21.53 (3.62), 19.57 (5.20) respectively. For fatigue impact, the mean (SD) MFIS score of both groups were 13.17 (3.81) and 12.69 (3.89) at baseline and 9.00 (3.75) and 12.88 (3.89) at post-treatment, respectively. The authors concluded that the CBT group had significantly lower scores on both the fatigue scale and the MFIS compared with the control group. This study was limited by being a pilot/feasibility trial and recruitment occurred through the Internet which may decrease the validity of the study.

Nazari et al. (35) undertook a single-blinded randomized controlled clinical trial aiming to compare the effects of reflexology and relaxation on fatigue in women with MS. A total of 75 participants were randomly assigned to three groups: reflexology, relaxation, and control groups (25 PwMS per group). The intervention of relaxation was performed for 4 weeks (twice a week for 40 min in each session). Outcome assessments were done before, immediately after, and 2 months after intervention. The FSS was used for fatigue measurement. The author reported a significant difference was found in the fatigue mean scores in all three measurements (before, immediately after, and 2 months after intervention) of the relaxation group [*P*-value < 0.001 as reported by Nazari et al. (35)]. The mean fatigue severity scores immediately after intervention was significantly lower in relaxation group than the control group [*P*-value = 0.01 as reported by Nazari et al. (35)]. Limitations of this study were lack of blinding to participants, use of simple, non-random sampling before random assignment into three groups, and lack of information about follow-up rates. Since participants were aware of their related arm, contamination among participants may have been possible. Although participants were asked not to use the technique alone at home until the end of the study, there was no monitoring to establish whether or not this occurred. These biases may have affected the validity of the study.

Spitzer and Pakenham (54) presented a pilot study with single-group pre-test/post-test study design with the aim to evaluate a community-based mindfulness intervention. Twenty-three PwMS received a mindfulness program for 5 weeks (one 2-h session per week). The fatigue assessments were taken at pre-intervention, post-intervention, and 8-week follow-up using the MFIS. The mean (SD) of MFIS was 2.32 (0.90) at pre-intervention, 2.17 (0.73) at post-intervention, and 2.33 (0.77) at follow-up. The authors reported that the mindfulness intervention had no significant impact on fatigue. The validity of the results of the study may have been limited by the small sample size, non-random sampling, and reliance on self-report including MS course and diagnosis, and lack of information about blinding.

Thomas et al. (47, 48) carried out a multi-center RCT to assess the effectiveness of a group-based fatigue management program (FACETS) for PwMS. The 164 participants were randomly allocated into two groups (84 participants in FACETS and 80 participants in a control group—current local practice). The FACETS program is mainly based on CBT and energy effectiveness techniques. Two experienced health professionals delivered the FACETS program to groups of 6–12 participants over six weekly 90-min sessions. Fatigue severity was measured at baseline, 1 month, 4 months, and 12 months after the final FACETS session. The study reported less fatigue for those undertaking the FACETS program at the 4- and 12-month follow-up (change from baseline = −0.36; 95% CI: −0.63 to −0.08; *P*-value = 0.01 at 4-month follow-up; change from baseline = −0.30; 95% CI: −0.61 to 0.01; *P*-value = 0.06 at 12-month follow-up). Lack of information about treatment fidelity across different centers and participants knowing their interventions may have affected the validity of this study.

van Kessel et al. (49) presented a RCT to assess the efficacy of CBT for MS-related fatigue. A total of 72 PwMS were randomly allocated to receive eight weekly sessions of CBT (35 participants) or relaxation training (RT) (37 participants). Self-rated outcome measures were collected at four points (pre- and post-treatment, 3 months and 6 months after post-treatment). Fatigue was measured by using the Fatigue Scale. In this study, a normative approach was used and fatigue scale data for a matched-healthy group (72 healthy participants) were collected during the baseline assessment. According to the findings, in both groups, there were reductions in fatigue at post-treatment and follow-up. In addition, the author mentioned that the CBT group showed significantly greater reductions in fatigue level than the RT-group at the end of treatment and follow-up periods [*P*-value < 0.02—as reported by van Kessel et al. (49)]. At the end of treatment, the CBT group showed a significantly lower level of fatigue compared with the healthy comparison group [*P*-value < 0.001—as reported by van Kessel et al. (49)] and fatigue levels of the RT group were equivalent to those of the matched-healthy group. It was also difficult to determine the validity of the results of this study as the authors did not provide details regarding the blinding of assessors and participants.

van Kessel et al. (55) described a pilot RCT to compare the efficacy of a web-based CBT self-management program with and without the use of email support (therapeutic contact). A total of 39 PwMS were randomly allocated into either an Internet-based CBT with email support from a clinical psychologist (MSInvigor8-Plus) or an Internet-based CBT without email support (MSInvigor8-Only). MSInvigor8 included eight sessions of between 25 and 50 min. The outcome measures included fatigue severity (Fatigue Scale) and impact (MFIS) and were conducted at baseline and at 10 weeks. The authors found that the MSInvigor8-Plus intervention significantly reduced in fatigue severity [*P*-value < 0.01—as reported by van Kessel et al. (55)] and fatigue impact [*P*-value < 0.02—as reported by van Kessel et al. (55)] when compared with the MSInvigor8-Only group. In this study, there was no blinding for group allocation of participants and researchers. A Hawthorne effect could have existed since the participants knew their allocated interventions. Furthermore, only participants who were interested and able to use Internet-based CBT were included in this pilot RCT and only 45% of the MSInvigor8-Only group completed post-treatment follow-up. These limitations of the study may decrease the validity of the findings.

Vazirinejad et al. (50) presented a placebo-controlled clinical trial to evaluate the effectiveness of psychological training with gradual muscle relaxation technique on fatigue in PwMS. A total of 60 participants were randomly allocated to two groups: intervention and control group (no intervention) (30 PwMS per group). The intervention group received 12 sessions of psychological training with gradual muscle relaxation technique (2 sessions per week). Outcome assessments were done before, immediately after, and 3 months following intervention. The FSS was used for fatigue measurement. According to the finding, the means of intervention group were 42.83 (8.36) at baseline and 33.9 (7.07) at immediately after the intervention. The authors found a significant reduction in the FSS in the intervention group [*P*-value ≤ 0.001—as reported by Vazirinejad et al. (50)]. No information about randomization, blinding, and follow-up rate was provided in this study. Therefore, it is difficult to determine the validity of estimates.

### Meta-Analyses

There were 12 studies (35, 39–50) (*n* = 745) included in 4 meta-analyses. In the first meta-analysis, five studies (41–43, 46–48) (*n* = 429) compared CBT against non-active controls (i.e., waitlist, standard care, or current local practice). In the second meta-analysis, three studies (44, 45, 49) (*n* = 141) compared CBT interventions or SMOoTh (CBT) against active controls (i.e., relaxation therapy or SEGP). In the third meta-analysis, two studies (35, 50) (*n* = 110) compared relaxation therapy against non-active controls (routine treatment or no intervention) and in the fourth meta-analysis, two studies (39, 40) (*n* = 65) compared mindfulness intervention against non-active controls (i.e., waitlist group).

#### Efficacy of Psychological Interventions (CBT) Against Non-Active Controls on MS-Related Fatigue (Number of Studies = 5, *n* = 429)

**Figure 2:**
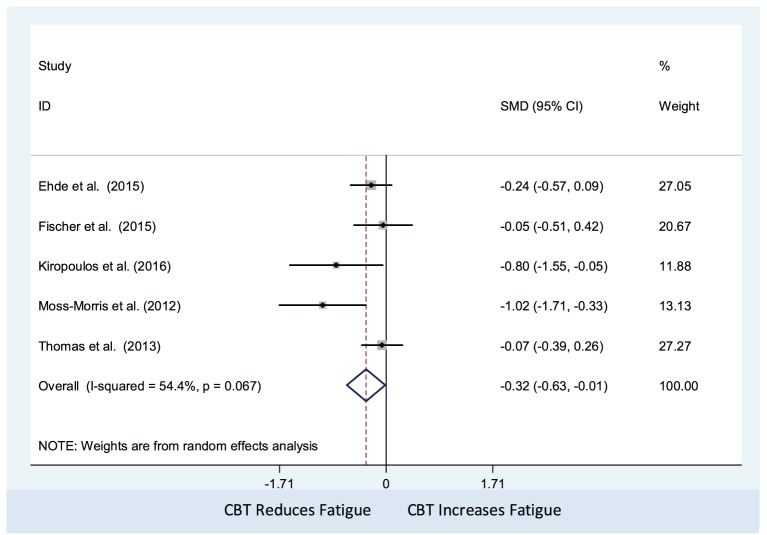
Comparison of cognitive behavioral therapy interventions and non-active controls on multiple sclerosis-related fatigue.

**Figure 3:**
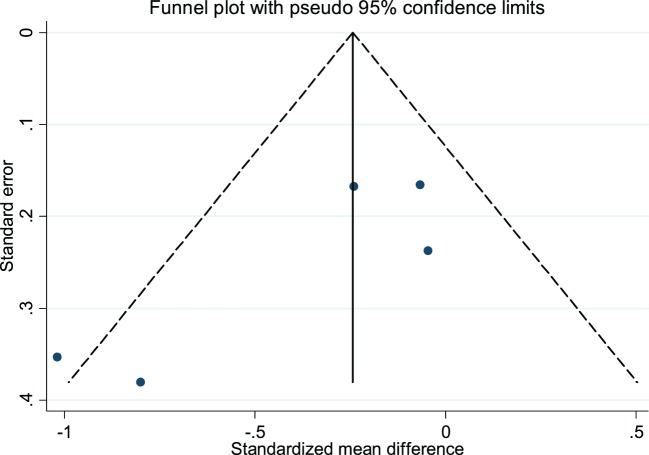
Funnel plot with pseudo 95% confidence limits for the meta-analysis of studies that reported a comparison of cognitive behavioral therapy interventions and non-active controls on multiple sclerosis-related fatigue. Solid vertical lines correspond to pooled standardized mean difference (SMD), dotted lines corresponds to the pseudo 95% confidence limits, and solid dots correspond to each of the SMD from the included five studies.

The CBT intervention was associated with decreased fatigue (pooled SMD: −0.32; 95% CI: −0.63 to −0.01). There was moderate heterogeneity between studies (*I*^2^ = 54.4%; *P*-value = 0.07) (Figure 2). Visual inspection of the funnel plot did not indicate presence of small study effects (Figure 3) and Egger’s regression asymmetry test did not suggest any small study effects (*P*-value = 0.09).

#### Efficacy of Psychological Interventions (CBT) Against Active Controls on MS-Related Fatigue (Number of Studies = 3, *n* = 141)

**Figure 4:**
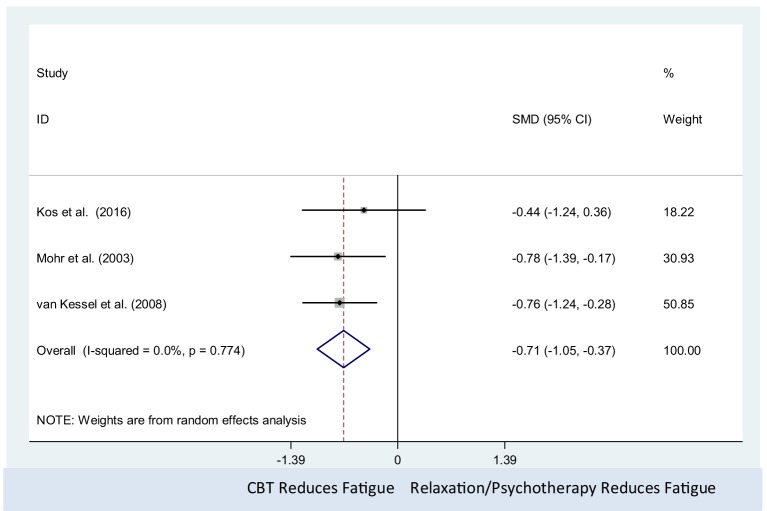
Comparison of cognitive behavioral therapy interventions and active controls (relaxation or psychotherapy) on multiple sclerosis-related fatigue.

The CBT intervention was associated with decreased fatigue (pooled SMD = −0.71, 95% CI: −1.05 to −0.37). There was no observed statistical heterogeneity between the three studies (*I*^2^ = 0%; *P*-value = 0.77) (Figure 4). Only three studies were included in this meta-analysis, which precluded us from assessing the presence of small study effects using funnel plots or Egger’s test (62).

#### Efficacy of Psychological Interventions (Relaxation Therapy) Against Non-Active Controls on MS-Related Fatigue (Number of Studies = 2, *n* = 110)

**Figure 5:**
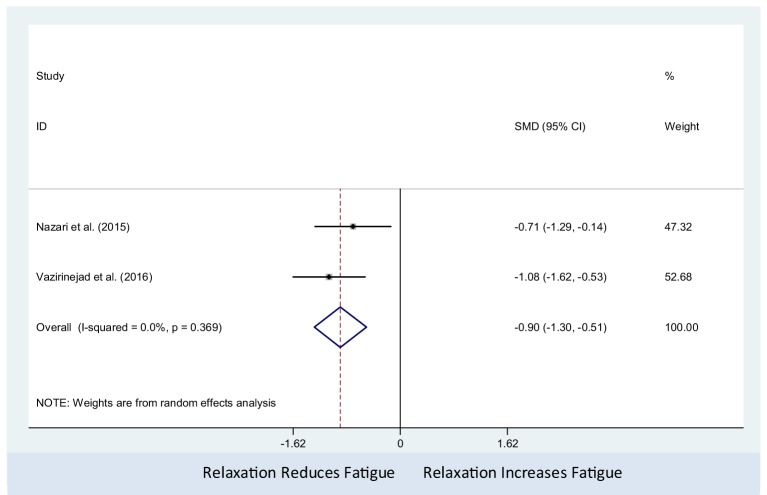
Comparison of relaxation and non-active controls on multiple sclerosis-related fatigue.

The relaxation therapy was associated with decreased fatigue (pooled SMD: −0.90; 95% CI: −1.30 to −0.51). There was no observed statistical heterogeneity between studies (*I*^2^ = 0%; *P*-value = 0.37) (Figure 5). Only two studies were included in this meta-analysis, which precluded us from assessing the presence of small study effects using funnel plots or Egger’s test (62).

#### Efficacy of Psychological Interventions (Mindfulness Interventions) Against Non-Active Controls on MS-Related Fatigue (Number of Studies = 2, *n* = 65)

**Figure 6:**
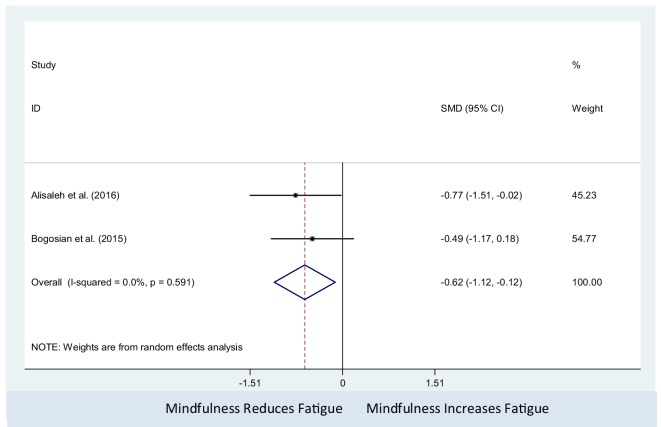
Comparison of mindfulness and non-active controls on multiple sclerosis-related fatigue.

The mindfulness intervention was associated with decrease in fatigue level (pooled SMD: −0.62; 95% CI: −1.12 to −0.12). There was no observed statistical heterogeneity between studies (*I*^2^ = 0%; *P*-value = 0.59) (Figure 6). Only two studies were included in this meta-analysis, which precluded us from assessing the presence of small study effects using funnel plots or Egger’s test (62).

## Discussion

The aim of this systematic review and meta-analysis was to determine the efficacy of psychological interventions in improving fatigue in PwMS. While others have undertaken a review (36) of the effect of CBT on fatigue in PwMS, our systematic review takes a broader perspective, needed to inform clinical guidelines. This review investigated 20 studies (22 articles) (33–35, 39–57) which assessed the following psychological interventions: CBT and CBT-related psychological treatment, relaxation, mindfulness, psychological training, intensive social cognitive wellness program, and progressive muscle relaxation. We include the studies which were excluded from the meta-analysis in this review since our focus was to inform the more comprehensive view of psychological interventions in MS-related fatigue management. Results of our narrative synthesis and meta-analysis suggest that psychological interventions, particularly CBT, relaxation therapy, mindfulness, and progressive muscle relaxation, are associated with reduced fatigue in PwMS. Interestingly, our meta-analysis of three papers (44, 45, 49) indicated CBT was most effective in reducing fatigue when compared with active controls (relaxation or SEGP). It should be noted that for all studies reviewed there was no differentiation of types of fatigue, i.e., whether fatigue was considered to be primary or secondary fatigue. In addition, Asano and Finlayson (30) undertook meta-analysis of three types of fatigue management interventions for PwMS. In the study of Asano and Finlayson, they used the label “education” to describe CBT, relaxation therapy, mindfulness therapy, and energy conservative course which was not the interest of our review and grouped all educational intervention together in one meta-analysis. In this review, we included only psychological interventions and performed separate meta-analysis for different types of psychological interventions.

### Summary of Main Findings

#### Cognitive Behavioral Therapy

This review suggests that CBT is an effective psychological method of treating MS-related fatigue. Among the 20 studies included in this review, in total, 7 studies (41–43, 46–48, 51, 55) delivered CBT as a psychological intervention for fatigue treatment among PwMS. Among them, five studies (41–43, 46–48) examined the effect of CBT against non-active controls such as telephone-delivered education intervention, waitlist, current local practices, and standard care, whereas Anderson et al. (51) assessed the efficacy of CBT through the single group pre-test and post-test study design and van Kessel et al. (55) compared the effect of a web-based CBT with or without email support from clinical psychologists. In all seven studies, CBT decreased the fatigue level of PwMS. In the study of Ehde et al. (41), Kiropoulos et al. (43), Moss-Morris et al. (46), and Thomas et al. (47, 48), the authors concluded that CBT resulted in clinically significant decrease in MS-associated fatigue and was effective in managing fatigue. In the study of van Kessel et al. (55), the Internet-based CBT with an email support from a skilled clinical psychologist had significantly greater reduction in fatigue level compared with the Internet-based CBT without an email support.

The results of our meta-analysis of five studies (41–43, 46–48) indicated that CBT, compared with non-active control, had a significant effect in reducing MS-related fatigue and we found moderate heterogeneity between studies. This might be associated with the ways in which CBT was delivered, as these were slightly different across the studies. In the study of Ehde et al. (41), the intervention was a telephone-delivered self-management program consisting of CBT and positive psychology methods, whereas in the pilot RCT of Moss-Morris et al. (46), the authors used an Internet-based CBT self-management program. By contrast, Fischer et al. (42) used an online program based on principles of CBT with a focus on reducing depression, and Kiropoulous et al. (43) delivered a tailored CBT-based intervention for depressive symptoms among newly diagnosed PwMS. In the trial by Thomas et al. (47, 48), the intervention was a group-based fatigue management session in which CBT components were dominant.

#### CBT Compared With Active Controls

The trials of Kos et al. (44), van Kessel et al. (49), and Mohr et al. (45) investigated the effect of CBT against active controls, which were relaxation therapy or psychotherapy. Three studies showed CBT compared with active controls was effective in treating fatigue. Among the three studies (44, 45, 49) which compared CBT against active controls such as relaxation therapy in fatigue treatment, van Kessel et al.’s study (49) and Mohr et al.’s study (45) demonstrated that both CBT and active controls (relaxation therapy or psychotherapy) showed clinically significant decreases in MS-associated fatigue. The meta-analysis revealed that CBT produced statistically significant decreases in fatigue level. Therefore, we can conclude that CBT is more effective for fatigue treatment compared with active controls such as relaxation therapy and SEGP, this is in line with the findings from a meta-analysis conducted in 2016 by van den Akker (36). van den Akker found that the use of CBT had a moderately positive effect on MS-related fatigue management. There are differences between the study of van den Akker et al. (36) and the present study. The review of van den Akker et al. focused exclusively on CBT and included only RCTs while our review assessed a wider range of psychological interventions; and included a single-group pre-test/post-test design study, RCTs, and randomized clinical trials.

#### Relaxation, Mindfulness, Muscle Relaxation Technique, and Intensive Social Cognitive Treatment

Three studies (35, 50, 52) delivered relaxation therapy and psychological training for PwMS. Across the three studies, it was found that relaxation and psychological training was significantly effective in reducing fatigue. In Carletto et al.’s study (52), the psychological interventions (relaxation therapy and EMDR) were designed to treat post-traumatic stress disorder in PwMS, whereas Nazari et al. (35) and Vazirinejad et al. (50) delivered relaxation and psychological training with gradual muscle relaxation technique for MS-related fatigue treatment. The results of our meta-analysis of two studies (35, 50) revealed that relaxation therapy, compared with non-active control, had a significant effect in reducing MS-related fatigue.

Furthermore, in our study, a total of four studies (39, 40, 53, 54) used mindfulness interventions as psychological interventions for PwMS. In all four studies, mindfulness interventions decreased the fatigue of PwMS. However, only two studies, Alisaleh and Shahrbanoo (39) and Grossman et al. (53) showed that mindfulness interventions were significantly effective in reducing fatigue. The results of our meta-analysis of two studies (39, 40) showed that mindfulness intervention, compared with non-active control, had a significant effect in reducing fatigue among PwMS.

Moreover, this review included one study (33) which examined the effect of PMRT through a single-group pre-test/post-test design study. In that study, PMRT significantly decreased MS-related fatigue. In the trial conducted by Mackay et al. (34), RMSSE were delivered to both intervention and control groups and biofeedback was applied to the intervention arm. Both groups showed reduction in fatigue levels, but a statistically significant difference was found only in the intervention group. In addition, our systematic review included one observational study (56, 57) in which the effect of an intensive social cognitive wellness program on fatigue of PwMS was examined; however, no effect on fatigue was found.

### Interpretation

Across the studies included in this review, the characteristics of participants were reasonably homogeneous. It is unlikely therefore that the findings observed can be attributed to differences in participant’s characteristics. The fatigue scales used by the studies reviewed, were not consistent between studies and the nature of each fatigue measurement scale were also slightly different. The FSS measures the severity of fatigue and its effect on a person’s activities and lifestyle (14). The FSS has moderate reliability (ICC = 0.751) (63), concurrent validity (Cronbach’s alpha = 0.86) (64), high internal consistency (Cronbach’s alpha 0.95) (65), and it correlates well with MFIS (*r* = 0.754) (63). The MFIS provides an assessment of the effects of fatigue in terms of physical, cognitive, and psychosocial functioning (15). The global fatigue severity subscale of the FAI is the tool that is used to differentiate normal fatigue from fatigue by medical disorders (66). The FSMC is a patient reported outcome measure for measuring mental and physical fatigue, and it is the scale with high internal consistency (Cronbach’s alpha > 0.91) that was tested against other fatigue scales and provide graduation of cognitive and motor fatigue (67). The fatigue scale measures the severity of physical and mental fatigue, and it has high degree of internal consistency and the validation coefficients were sensitivity 75.5 and specificity 74.5 (68). Clearly, it is likely therefore that these tools measure different aspect of fatigue and are not directly comparable. This should be considered when interpreting results of this systematic review. While this can be accounted for to some extent in meta-analysis using SMD, the use of different scales makes comparison between studies included in our narrative review difficult.

The psychological interventions used in the included studies were also quite varied. In the trials of Mohr et al. (45), Kos et al. (44), and van Kessel et al. (49), CBT and self-management therapy (CBT) were used as psychological interventions and the effects of psychological treatments in improving MS-related fatigue were compared against an active-control such as relaxation therapy and SEGP which were also the psychological interventions. Due to this design, the effect of CBT might be underestimated. In the study of Thomas et al. (47, 48), the authors used a pragmatic parallel arm multi-center RCT to investigate the effect of a group-based fatigue management intervention which was mainly based on CBT, but treatment fidelity between centers was not formally assessed. In five studies (39, 41–43, 45, 53), psychological interventions were used separately for reducing not only MS-associated fatigue but also depression or stress. Therefore, the intervention approaches of these six studies might be slightly different in comparison with other trials which were focused on fatigue treatment.

In addition, with respect to follow-up rates and attrition rates of all included studies in this review, approximately three-fourths (33, 40–44, 46–49, 51–57) of included studies reported follow-up rates in detail. Attrition across the included studies except van Kessel et al. (55) was low, with only 45% of the control group completed follow-up with unknown reasons.

### Strengths of the Review

This review employed a rigorous methodological strategy to search and appraise the literature. We used a broad search strategy that was developed in consultation with a librarian, and hand-searched relevant systematic review to identify relevant studies. Two reviewers were independently involved in the screening, data extraction, and appraisal of studies suitable for inclusion. When required, disagreements were resolved by further consensus with authors Tracey J. Weiland and Alysha M. De Livera. Two reviewers independently assessed the quality of included studies in accordance with the Effective Public Health Practice Project Quality Assessment Tool for Quantitative Studies (Hamilton Tool) (38). We also contacted primary authors for missing information or required data for our narrative review and meta-analyses.

### Limitations of the Review

We limited included articles to those published in English which might have excluded relevant studies published in other languages. As well, only 12 studies (35, 39–50) were included in the meta-analyses. In addition, the follow-up lengths of the included studies were inconsistent and most of them were not longer than 12 months. Therefore, no meta-analysis was conducted to investigate the long-term effectiveness of psychological interventions. Finally, since there were fewer than 10 studies included in the meta-analysis, caution should be taken when interpreting the results from Egger’s test (62).

### Conclusion

The finding of this review can be used to inform practice as well on clinical recommendations for psychological approaches in MS-related fatigue management. Psychological interventions such as CBT, mindfulness-based therapy, and relaxation therapy were effective in the treatment of MS-related fatigue. Most importantly, CBT was more effective in reducing fatigue levels compared with other psychological interventions such as relaxation therapy and supportive-expressive group psychotherapy. More studies are needed to investigate the efficacy of each of mindfulness, relaxation therapy, and progressive muscle relaxation for fatigue treatment; and compare the effectiveness of these kinds of psychological interventions with CBT in fatigue management. A thorough examination of the convergent validity of the fatigue scales is also warranted. Furthermore, an exploration of the long-term effect of all types of psychological interventions for fatigue management for PwMS is needed.

## Author Contributions

AZZP contributed to study design, undertook searches, review of abstracts and papers, quality appraisal, meta-analyses, and drafted the manuscript. TD contributed to review of abstracts and papers for relevance, and quality appraisal, and reviewed and approved the final manuscript. AL contributed to study design, supervised meta-analyses, and edited the final manuscript. GJ, CB, CM, SN, KT, TM, AK, and EO’K contributed to interpretation and editing of the final manuscript. EO’K provided advice on meta-analyses and edited the final manuscript. TW conceived the project and provided overall supervision for the project, and edited and approved the final manuscript.

## Conflict of Interest Statement

GJ receives royalties from the book “Overcoming Multiple Sclerosis,” and has received remuneration for running overcoming MS retreats, and is Chief Editor for Frontiers in Neurology (Neuroepidemiology Section); SN and KT have received remuneration for running overcoming multiple sclerosis retreats, and are both review editors for Frontiers in Neurology; TW is an associate editor for Frontiers in Neurology (Neuroepidemiology Speciality); CM is a review editor for Frontiers in Neurology (Neuroepidemiology Speciality).

## Abbreviations

CBT, cognitive behavioral therapy; EPHPP, Effective Public Health Practice Project; FACETS, fatigue: applying cognitive behavioral and energy effectiveness techniques to lifestyle; FSMC, fatigue scale for motor and cognitive function; FSS, Fatigue Severity Scale; MBI, mindfulness-based intervention; MFIS, Modified Fatigue Impact Scale; PMRT, progressive muscle relaxation technique; PRISMA, preferred reporting items for systematic reviews and meta-analyses; PwMS, people with MS; RMSSE, relaxation, mindfulness, social support, and education; RT, relaxation training; SMOoTh, self-management occupational therapy; T-SM, telephone-delivered self-management intervention; T-ED, telephone-delivered parallel education; UC, usual care.

## Appendix

**Table A1:** Search strategy.

Literature search, April 5, 2017	
Medline (Ovid)	Multiple sclerosis (MS) Fatigue or energy or lassitude Cognitive behavio* therapy or CBT or psychological therapy or stress reduction technique* or meditation or mindfulness or relaxation or guided imagery or progressive muscle relaxation or educational counseling 1 and 2 and 3

EMBASE (Ovid)	MS Fatigue or Energy or Lassitude Cognitive behavio* therapy or CBT or psychological therapy or stress reduction technique* or meditation or mindfulness or relaxation or guided imagery or progressive muscle relaxation or educational counseling 1 and 2 and 3

PsycINFO (Ovid)	MS Fatigue or Energy or Lassitude Cognitive behavio* therapy or CBT or psychological therapy or stress reduction technique* or meditation or mindfulness or relaxation or guided imagery or progressive muscle relaxation or educational counseling 1 and 2 and 3

CINAHL	“MS” Fatigue or Energy or Lassitude “Cognitive behavio* therapy” or CBT or “psychological therapy” or “stress reduction technique*” or meditation or mindfulness or relaxation or “guided imagery” or “progressive muscle relaxation” or “educational counseling” 1 and 2 and 3

**Table A2:** Articles excluded from the systematic review.

Reference	Reasons for exclusion from systematic review
Askey-Jones et al. (69)	Multicomponent interventions that did not isolate psychological therapy

Bisht et al. (70)	The study did not include a psychological intervention

Burschka et al. (71)	Intervention (Tai Chi) was not a psychological intervention

Cosio et al. (72)	Outcome of the study was quality of life (no fatigue outcome)

Finlayson et al. (73)	Intervention was predominantly education about fatigue

Kinsinger et al. (74)	There was no separate outcome data for the two interventions

Knoop et al. (75)	There was no comparison of fatigue with control. Comparison issue

Kos et al. (76)	This study was not predominantly psychological interventions, but did include relaxation

Levin et al. (77)	No fatigue outcome data was reported

Mohr et al. (78)	No comparison data

Nejati et al. (79)	Separate data was not reported for mindfulness and yoga

Tesar et al. (80)	The intervention of this study was neurorehabilitation

Thomas et al. (81)	Qualitative study of 16 people

**Table A3:** Studies excluded from the meta-analysis.

Reference	Main reasons for excluding study from the meta-analysis
Anderson et al. (51)	No control comparison Single-group pre- and post-test study design

Carletto et al. (52)	Solitary study with a comparison of relaxation and active control (eye movement desensitization and reprocessing) which was the intervention of the Carletto et al.’s study

Dayapoglu and Tan (33)	No control comparison Single-group pre-test and post-test study design

Grossman et al. (53)	Insufficient data to include in the meta-analysis and the authors were unable to supply the requested data information

Jongen et al. (56, 57)	No control comparison

Mackay et al. (34)	Both groups received relaxation, mindfulness, social support, and education programs and the only difference between the groups was biofeedback which was not the focus of this review

Spitzer and Pakenham (54)	No control comparison Single-group pre-test and post-test study design

van Kessel et al. (55)	Both intervention groups received CBT and the only difference between the groups was with or without email support from a clinical psychologist
